# The Relationship Between Clinical Outcomes and Calculated Thrombus Burden Before and After Initial Flow in Patients with ST-Segment Elevation Myocardial Infarction

**DOI:** 10.5152/eurasianjmed.2022.21021

**Published:** 2022-06-01

**Authors:** Oğuzhan Birdal, Selim Topçu, İbrahim Halil Tanboğa, Yavuzer Koza, Emrah Aksakal, Oktay Gülcü, Şule Karakelleoğlu, Hüseyin Şenocak, Fuat Gündoğdu

**Affiliations:** 1 Department of Cardiology, Atatürk University Faculty of Medicine, Erzurum, Turkey; 2 Erzurum Training and Research Hospital, Erzurum, Turkey

**Keywords:** Myocardial infarction, thrombus burden, no-reflow

## Abstract

**Objective:** Primary percutaneous coronary intervention is the standard treatment for ST-segment elevation myocardial infarction. Although myocardial and epicardial perfusion is usually achieved with primary percutaneous coronary intervention, infarct-related arterial thrombus burden negatively affects the procedural success and clinical outcomes of primary percutaneous coronary intervention. Therefore, we aimed to investigate the association between thrombus burden (calculated before and after initial flow) and clinical consequences in patients with ST-segment elevation myocardial infarction.

**Materials and Methods:** This study retrospectively enrolled 1376 patients who had ST-segment elevation myocardial infarction between May 2012 and November 2015. Patients who had only undergone balloon angioplasty and emergency coronary artery bypass grafting were not included in the study. Data regarding the initial clinical and demographic features of the patients were obtained from their hospital records. Thrombus burden was calculated using baseline and final (after wire inflation or small balloon dilatation) thrombolysis in myocardial infarction thrombus grades. The endpoints of the study were defined as no-reflow development after primary percutaneous coronary intervention and 1-year all-cause mortality. Statistical significance was defined as *P* < .05.

**Results:** No-reflow was detected in 169 patients (12.3%). The calculated basal thrombus burden was significantly associated with post-procedural no-reflow (*P* < .001). No-reflow was also associated with advanced age (*P* < .001), longer pain-to-door time (*P* < .001), and increased blood glucose levels (*P* = .032). The calculated final thrombus burden was related to 1-year all-cause mortality (*P* = .047). One-year all-cause mortality was also associated with advanced age (*P* < .001), high Killip scores (*P* = .003), increased white blood cell counts (*P* = .001), and low estimated glomerular filtration rates (*P* < .001).

**Conclusion:** Basal thrombus burden was associated with no-reflow, and final thrombus burden was associated with 1-year all-cause mortality. The calculation of thrombus burden before and after initial flow may help to predict clinical outcomes.

## Introduction

Most cases of ST-segment elevation myocardial infarction (STEMI) result from an atherosclerotic plaque rupture which leads to the formation of an intraluminal occlusive thrombus. Primary percutaneous coronary intervention (PPCI)—the standard treatment for STEMI—is used to achieve rapid coronary reperfusion. However, in some cases, PPCI may not provide adequate epicardial and myocardial perfusion.^1,2^

Several studies have proposed that thrombus burden (TB) may contribute to inadequate epicardial/myocardial perfusion; therefore, these studies encouraged the use of thrombus aspiration (TA).^3-5^ However, the TA in STEMI in Scandinavia (TASTE) trial—the first large study of this treatment in STEMI patients—demonstrated that routine TA was not beneficial for patients with STEMI with regards to 1-year clinical outcomes.^6,7^ Furthermore, the subgroup analysis of the TASTE trial showed that the risks of clinical outcomes were similar in cases with low versus high TB. In this trial, TB was assessed using the thrombolysis in myocardial infarction (TIMI) thrombus scale after wiring and/or small balloon inflation, showing that a relatively low number of patients (30%) had high TB. Thrombus burden after wiring and/or small balloon inflation was associated with neutral effects of TA on clinical outcomes.

The trial of routine aspiration thrombectomy with PCI versus PCI alone in patients with STEMI (TOTAL) remains the largest study to date. Similar to the TASTE trial, TOTAL revealed that TA had no effect on clinical consequences.^8^ Moreover, as in the TASTE trial, there were no remarkable differences in clinical outcomes between the subgroups with low and high TB. In the TOTAL trial, TIMI thrombus scale was used; however, unlike the TASTE trial, the TOTAL trial only calculated TB based on an initial diagnostic angiogram.

In the present study, we hypothesized that TB calculated based on an initial angiogram or after wiring/small balloon inflation may be associated with certain clinical outcomes. Therefore, we used the TIMI scale to calculate TB based on an initial angiogram and after wiring/small balloon inflation. Then, we investigated the associations between TIMI TB and 1-year clinical outcomes in STEMI patients who underwent PPCI.

## Materials and Methods

### Study Population

The present study retrospectively enrolled 1376 patients with STEMI who had undergone PPCI between May 2012 and November 2015. The inclusion criteria were as follows:

- patients presenting within the first 12 hours after the onset of chest pain (18 hours for cardiogenic shock);
- an ST elevation of at least 1 mm in 2 or more contiguous leads (2 mm for leads V1-V3) or a new-onset left bundle branch block.

Patients who had only undergone balloon angioplasty and emergency coronary artery bypass grafting (CABG) were excluded from the study. The study was approved by the Ethics Committee of Atatürk University (05.05.2017, 2/22). Written informed consent was obtained from all participants who participated in this study.

A 300 mg of aspirin and a loading dose of 600 mg of clopidogrel or 180 mg of ticagrelor were administered to all patients at initial admission. Intravenous unfractionated heparin was given prior to the procedure at a dose of 100 IU/kg (maximal dose = 10.000 IU or 60 IU/kg in patients who were treated with glycoprotein IIb/IIIa inhibitors, GPI). All PPCI procedures were performed by experienced cardiologists through femoral route. After the intervention, all patients continued to receive dual antiplatelet therapy with 100 mg of aspirin per day and 75 mg of clopidogrel per day or 90 mg of ticagrelor twice per day.

### Data Collection and Definitions

Data regarding the clinical and demographic features of the patients were obtained from their medical records. Laboratory analysis for fasting blood glucose, creatinine, troponin I, C-reactive protein (CRP), and complete blood count (white blood cell, WBC, and hemoglobin, Hgb) were performed in all patients upon admission. Blood samples for troponin I were remeasured every 6 hours daily up to peak levels were detected. The estimated glomerular filtration rate (eGFR) was measured using the Modification Diet in Renal Disease (MDRD) formula.^9^

### Angiographic Analysis of Thrombus Burden

**Figure 1. a-f.**
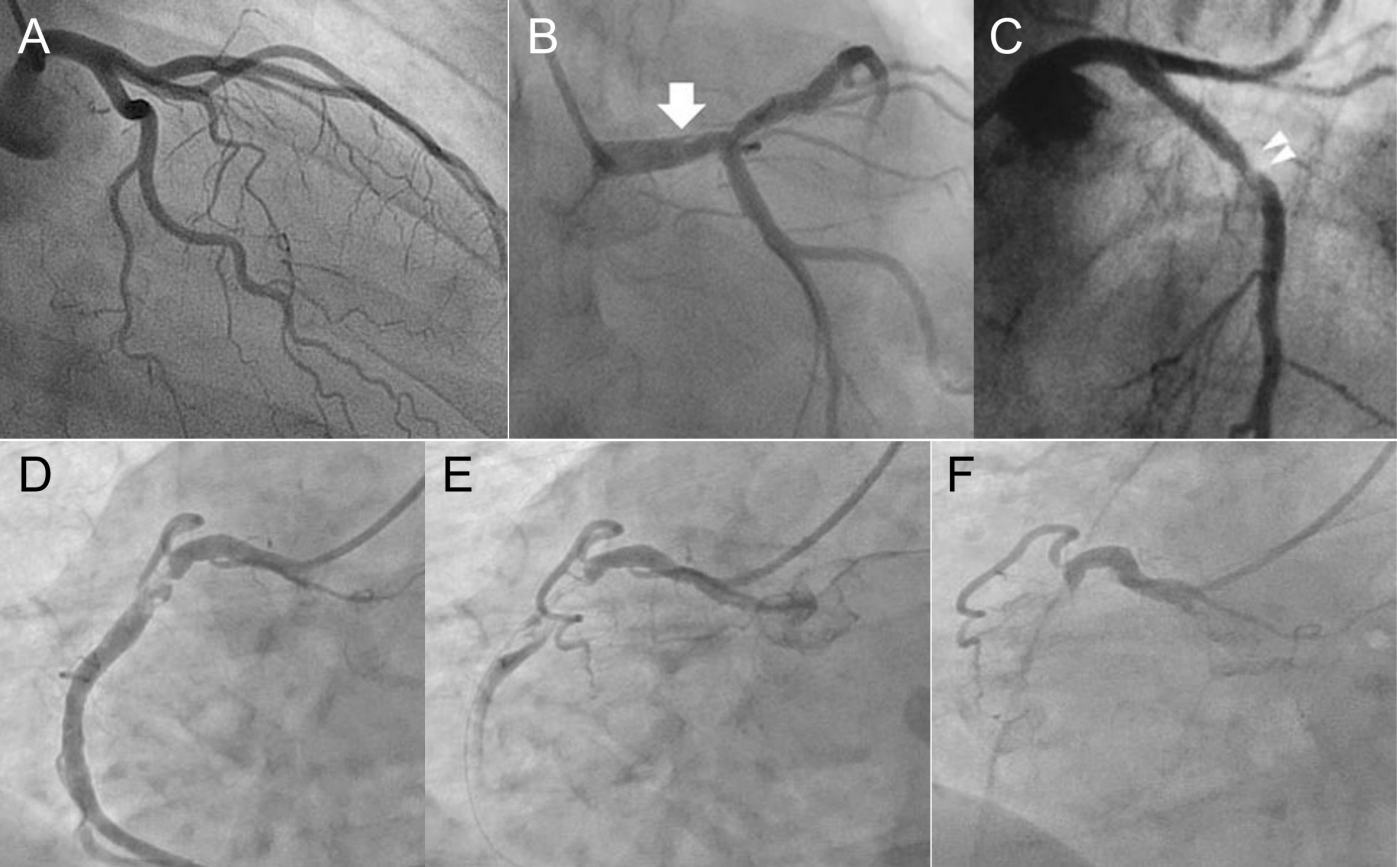
TIMI thrombus classification. (a) TIMI thrombus grade 0; (b) TIMI thrombus grade 1 (white arrow); (c) TIMI thrombus grade 2 (arrows); (d) TIMI thrombus grade 3; (e) TIMI thrombus grade 4; (f) TIMI thrombus grade 5. TIMI, thrombolysis in myocardial infarction.

No-reflow was defined as suboptimal myocardial flow despite PCI with stenting and/or ballooning.^10^ Angiographic coronary TB was categorized in 5 grades.^11^ Detailed examples of the TB classifications are presented in Figure 1. The TIMI thrombus grades were determined based on an initial coronary angiogram (initial TB) and immediately after antegrade flow was restored via an angioplasty guidewire or a small balloon dilation (using balloons with diameters of 1.5-2.5 mm) in patients with TIMI Thrombus Grade 5. The coronary angiogram allowed for regrading of the underlying residual thrombus (final TB; 3). Then, the final TIMI thrombus grades were stratified into classifications of low TB (Grades 0-3) and high TB (Grades 4-5; 12).

### Clinical Outcomes and Follow-Up

The endpoints of our study were defined as the development of no-reflow after PPCI and 1-year all-cause mortality. For the post-procedural angiography, no-reflow was described as TIMI 0/1/2 flow or myocardial blush grade (MBG) 0/1.^13,14^ All-cause mortality was determined using hospital recordings and TR identity numbers.

### Statistical Analysis

All statistical analyses were performed using Statistical Package for the Social Sciences software v.20 (IBM SPSS Corp.; Armonk, NY, USA). Numerical variables were expressed as means ± standard deviations, and categorical variables were expressed as percentages. Chi-square or Fisher’s exact tests were used to compare categorical variables, and Student’s *t* tests or Mann–Whitney *U* tests were used to compare numerical variables. A multivariate logistic regression analysis was performed to determine whether no-reflow and clinical outcomes were related to the following variables: age, pain door, door balloon time, white blood cell count, hemoglobin level, platelet level, eGFR, blood glucose level, lipid panel results, gender, diabetes mellitus, hypertension, systolic and diastolic blood pressures, smoking, MI type (anterior/inferior), history of previous PCI or CABG, and Killip class (1 or 2/3/4). A *P* value < .05 indicated statistical significance.

## Results

**Figure 2.**
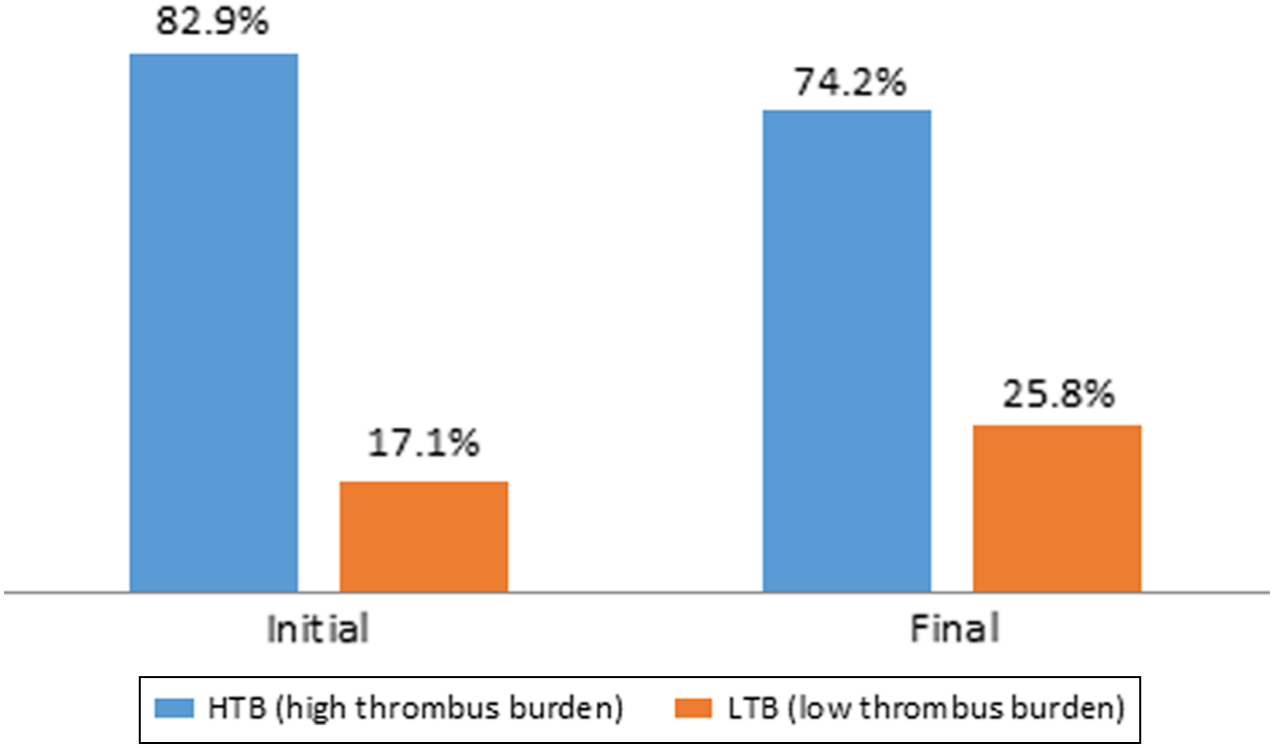
Initial and final thrombus burden.

A total of 1376 patients (58.4 ± 11.7 years of age, 76.2% male) with STEMI who underwent PPCI were included in this study. The baseline clinical features of the study population are presented in Table 1. The TB was recalculated after guidewiring or small balloon dilation for patients with TIMI Thrombus Grade 5. The initial and final TB distributions are presented in Table 2, and the percentages of low versus high initial and final TB are presented in Figure 2.

Examination of the patients’ post-procedure records revealed that no-reflow developed in 169 (12.3%) patients. The percentage of glycoprotein IIb/IIIa inhibitor usage was 43.6%. A univariate analysis showed that post-procedure no-reflow development was associated with advanced age, diabetes mellitus, hypertension, long pain door time, advanced Killip classes, high systolic and diastolic blood pressures, increased white blood cell and glucose levels, low eGFR, and high initial and final thrombus burden (Table 3). A multivariate logistic regression analysis showed that advanced age (odds ratio (OR): 1.04, 95% CI: 1.02-1.06, *P* < .001), long pain door time (OR: 1.005, 95% CI: 1.004-1.007, *P* < .001), high blood glucose (OR: 1.003, 95% CI: 1.000-1.005, *P* = .032), and high basal TB (OR: 11.8, 95% CI: 3.8-36.2, *P* < .001) were independent predictors of post-procedure no-reflow development (Table 4).

Furthermore, 95 patients (6.9%) had died of any cause by the 1-year follow-up. A univariate analysis showed that 1-year all-cause mortality was associated with advanced age, female gender, diabetes mellitus, hypertension, long pain door time, advanced Killip classes, high diastolic blood pressure, increased blood glucose, increased white blood cell count, low eGFR, low hemoglobin, increased platelets, and high final TB (Table 5). A multivariate logistic regression analysis revealed that advanced age (OR: 1.04, 95% CI: 1.02-1.06, *P* < .001), advanced Killip classes (OR: 2.36, 95% CI: 1.34-4.15, *P* = .003), high white blood cell count (OR: 1.139, 95% CI = 1.057-1.227, *P* = .001), low eGFR (OR: 0.978, 95% CI: 0.968-0.989, *P* < .001), and high final TB (OR: 1908, 95% CI: 1.008-3.612, *P* = .047) were independent predictors of 1-year all-cause mortality (Table 6).

## Discussion

In the present study, basal TB was associated with post-procedure no-reflow, and final TB was associated with 1-year all-cause mortality. Plaque rupture and the subsequent development of intracoronary thrombus play a central role in the pathogenesis of STEMI. Intracoronary thrombus impairs epicardial blood flow in both the pre-procedural and post-procedural periods and impairs myocardial perfusion in the post-procedural period due to an increased frequency of no-reflow and distal embolization.^3,5,15^ Several classification schemes are available to quantify intracoronary thrombus burden, such as the TIMI and Yip classification systems.^11,16^

Due to the associations between intracoronary TB and clinical outcomes, interest in the use of TA has increased significantly. Several small randomized, controlled trials have shown that TA is associated with remarkable improvements in epicardial/myocardial perfusion. The TA during percutaneous coronary intervention in acute myocardial infarction study (TAPAS) demonstrated that TA was associated with a lower frequency of adverse clinical consequences.^4^ Following this study, the use of TA significantly increased worldwide. Prior to the publication of the TASTE trial, no other TA trials had been conducted in patients with STEMI with adequate power. The TASTE trial—the first large, adequately powered trial of TA in STEMI patients—showed that routine TA did not reduce the rate or composite of all-cause mortality, stent thrombosis, or rehospitalization for myocardial infarction in patients with STEMI at the 1-year follow-up.^6,7^ More recently, the TOTAL study—the largest adequately powered study of TA in STEMI patients to date—showed that routine TA (vs. PCI alone) did not reduce the risk of recurrent myocardial infarction, cardiovascular death, NYHA class IV heart failure, or cardiogenic shock within 180 days in patients with STEMI who were undergoing PPCI.^8^ In addition, the subgroup analyses of both the TASTE and the TOTAL studies showed that TA did not improve clinical outcomes, even in patients with high TB.

Although the aforementioned studies of TA indirectly examined the negative effects of TB on clinical outcomes, small-scale TAPAS and thrombectomy with export catheter in infarct-related artery during PPCI (EXPIRA) studies have yielded some results in favor of TA.^4,17^ According to these results, TB has a negative impact on clinical outcomes. However, subsequent TASTE and TOTAL studies, which included approximately 18 000 patients in total (1071 of which were included in the TAPAS study), showed that TA did not improve clinical outcomes. A meta-analysis of 17 studies (20 960 patients), including the TASTE and TOTAL studies, concluded that TA has no clinical benefit and can lead to an increased risk of stroke.^18^ In the TASTE trial, TB was assessed using the TIMI scale and calculated after wiring and/or small balloon inflation. In both the TASTE and the TOTAL trials, clinical outcomes were similar in cases of low versus high TB. However, unlike the TASTE trial, the TOTAL trial calculated TB based on an initial diagnostic angiogram. In the present study, TB was calculated both at baseline and after wiring and/or small balloon inflation. The results of our study proposed that the negative effects of TB on clinical results were not detected in previous studies due to the calculation of TB at different stages of angiography.

Thrombus burden may be associated with adverse cardiac events, but TIMI-based thrombus classification may not be adequate in the quantification of TB. In this context, the TB classification system proposed by Yip et al^16^ may be more useful. Otherwise, a different classification system may be needed. However, there is not yet sufficient evidence in favor of TB classification systems other than the TIMI thrombus grade system.

The major findings of this study were that TB is significantly associated with no-reflow and 1-year mortality and should be calculated at different stages of angiography. Although current guidelines do not recommend it routinely, TA can be used in cases of high TB, especially in patients with isolated coronary thromboembolism.

The most important limitation of the present study was that it was conducted retrospectively. This limited the generalizability of the results. In addition, this study had a relatively low sample size compared to other large studies.

## Figures and Tables

**Table 1.** Baseline Patient Characteristics

**Age, years**	58.4 ± 11.7
**Gender (male), %**	76.2
**Diabetes mellitus, %**	22.8
**Hypertension, %**	42.8
**Dyslipidemia, %**	38.8
**Prior PCI, %**	11.8
**Prior CABG, %**	3.3
**Smoking, %**	54.7
**Anterior MI, %**	48
**Killip 2/3/4, %**	15.9
**Pain-to-door time, minutes**	180 ± 112
**Door-to-balloon time, minutes**	30 ± 6
**GPI, %**	43.6
**DES, %**	89.2

DES, drug eluting stent; GPI, glycoprotein IIb/IIIa inhibitors; CABG, coronary artery bypass graft.

**Table 2.** Distribution of Initial and Final Thrombus Burden After Recalculation of TIMI Thrombus Grade 5

	**Initial Thrombus Burden (%)**	**Final Grade 5 Thrombus Burden After Recalculation (%)**
**G0**	2 (0.1)	2 (0.1)
**G1**	11 (0.8)	11 (0.8)
**G2**	49 (3.6)	61 (4.4)
**G3**	174 (12.6)	281 (20.4)
**G4**	67 (4.9)	351 (25.5)
**G5**	1073 (78)	670 (48.7)

**Table 3.** Comparison of Clinical and Angiographic Features Between Groups With and Without No-Reflow

**Variables**	No-Reflow (+) (n = 169)	No-Reflow (−) (n = 1207)	*P*
**Age, years**	63.1 ± 11.7	57.8 ± 11.5	<.001
**Diabetes mellitus, %**	33.1	21.4	.001
**Smoking, %**	50.3	55.3	.217
**Dyslipidemia, %**	33.1	39.6	.106
**Anterior MI, %**	45.6	48.3	.504
**Pain-to-door time, minutes**	259 ± 131	169 ± 105	<.001
**Door-to-balloon time, minutes**	29.8 ± 5.7	29 ± 5.9	.514
**Killip 2/3/4, %**	27.2	15.1	<.001
**eGFR, mL/min/1.73 m** **2**	79 ± 26	88 ± 24.8	<.001
**Glucose, mg/dL**	182 ± 100	148 ± 70	<.001
**Initial thrombus burden (grades 4-5), %**	97.6	80.8	<.001
**Final thrombus burden (grades 4-5), %**	83.3	68.1	<.001

**Table 4.** Results of a Multivariate Logistic Regression Analysis Performed to Estimate Post-Processing No-Reflow

	**Univariate OR, 95% CI**	*P*	**Multivariate OR, 95% CI**	*P*
**Age**	1.040 (1.025-1.054)	<.001	1.04 (1.02-1.06)	<.001
**Pain-to-door time**	1.006 (1.005-1.007)	<.001	1.005 (1.004-1.007)	<.001
**Blood glucose level**	1.004 (1.003-1.006)	<.001	1.003 (1.000-1.005)	.032
**Initial thrombus burden**	9.8 (3.6-26.7)	<.001	11.8 (3.8-36.2)	<.001

OR, odds ratio.

**Table 5.** Comparison of Clinical and Angiographic Features Between Patients Who Had Died and Those Who Had Not Died by the 1-Year Follow-Up

**Variables**	Death (+) (n = 95)	Death (−) (n = 1281)	*P*
**Age, years**	66.8 ± 11.9	57.8 ± 11.4	<.001
**Gender (male), %**	40	22.6	<.001
**Diabetes mellitus, %**	45.3	42	<.001
**Smoking, %**	37.9	56	.001
**Killip 2/3/4, %**	46.3	14.4	<.001
White blood cell count, ×10**3** ** µL**	14.9 ± 5.3	12.1 ± 3.3	<.001
**Hemoglobin, g/dL**	12.5 ± 2.1	13.5 ± 1.9	<.001
Platelets, ×10**3** **µL**	289 ± 92	266 ± 66	.017
**eGFR, mL/min/1.73 m** **2**	64 ± 29	89 ± 24	<.001
**Glucose, mg/dL**	189 ± 96	149 ± 73	<.001
**Initial thrombus burden (grades 4-5), %**	88.4	82.4	.135
**Final thrombus burden (grades 4-5), %**	83.5	68.9	.003

eGFR, estimated glomerular filtration rate.

**Table 6.** Results of a Multivariate Logistic Regression Analysis Performed to Estimate 1-Year All-Cause Mortality

	**Univariate OR, 95% CI**	*P*	**Multivariate OR, 95% CI**	*P*
**Age, years**	1.069 (1.049-1.090)	<.001	1.055 (1.029-1.082)	<.001
**Killip 2/3/4**	5.144 (3.338-7.926)	<.001	2.36 (1.34-4.15)	.003
**White blood cell count**	1.191 (1.134-1.251)	<.001	1.139 (1.057-1.227)	.001
**eGFR**	0.960 (0.952-0.969)	<.001	0.978 (0.968-0.989)	<.001
**Final thrombus burden**	2.284 (1.295-4.029)	.004	1.908 (1.008-3.612)	.047

OR, odds ratio; eGFR, estimated glomerular filtration rate.

## References

[1] Ito H Maruyama A Iwakura K et al. Clinical implications of the ‘no reflow’ phenomenon. A predictor of complications and left ventricular remodeling in reperfused anterior wall myocardial infarction. Circulation. 1996;93(2):223 228. 10.1161/01.cir.93.2.223). 8548892

[2] White CJ Ramee SR Collins TJ et al. Coronary thrombi increase PTCA risk. Angioscopy as a clinical tool. Circulation. 1996;93:253 25 8. 8548896 10.1161/01.cir.93.2.253

[3] Sianos G Papafaklis MI Daemen J et al. Angiographic stent thrombosis after routine use of drug-eluting stents in ST-segment elevation myocardial infarction: the importance of thrombus burden. J Am Coll Cardiol. 2007;50(7):573 583. 10.1016/j.jacc.2007.04.059) 17692740

[4] Vlaar PJ Svilaas T van der Horst IC et al. Cardiac death and reinfarction after 1 year in the Thrombus Aspiration during Percutaneous coronary intervention in acute myocardial infarction Study (TAPAS): a 1-year follow-up study. Lancet. 2008 ;371(9628):1915 1920. 10.1016/S0140-6736(08)60833-8) 18539223

[5] Sianos G Papafaklis MI Serruys PW . Angiographic thrombus burden classification in patients with ST-segment elevation myocardial infarction treated with percutaneous coronary intervention. J Invasive Cardiol. 2010;22(10):6B 14B. 20947930

[6] . Lagerqvist B Frobert O Olivecrona GK , et al. Outcomes 1 year after thrombus aspiration for myocardial infarction. N Engl J Med. 2014;371:1111 11 20. 25176395 10.1056/NEJMoa1405707

[7] Fröbert O Lagerqvist B Olivecrona GK et al. Thrombus aspiration during ST-segment elevation myocardial infarction. N Engl J Med. 2013;369(17):1587 1597. 10.1056/NEJMoa1308789) 23991656

[8] Jolly SS Cairns JA Yusuf S et al. Randomized trial of primary PCI with or without routine manual thrombectomy. N Engl J Med. 2015;372(15):1389 1398. 10.1056/NEJMoa1415098) 25853743 PMC4995102

[9] Klahr S Levey AS Beck GJ et al. The effects of dietary protein restriction and blood-pressure control on the progression of chronic renal disease. Modification of diet in Renal Disease Study Group. N Engl J Med. 1994;330(13):877 884. 10.1056/NEJM199403313301301) 8114857

[10] Levine GN Bates ER Blankenship JC et al. 2015 ACC/AHA/SCAI focused update on primary percutaneous coronary intervention for patients with ST-elevation myocardial infarction: an update of the 2011 ACCF/AHA/SCAI guideline for percutaneous coronary intervention and the 2013 ACCF/AHA guideline for the management of ST-elevation myocardial infarction. J Am Coll Cardiol. 2016 ;67(10):1235 1250. 10.1016/j.jacc.2015.10.005) 26498666

[11] Gibson CM de Lemos JA Murphy SA et al. Combination therapy with abciximab reduces angiographically evident thrombus in acute myocardial infarction: a TIMI 14 substudy. Circulation. 2001;103(21):2550 2554. 10.1161/01.cir.103.21.2550) 11382722

[12] Niccoli G Spaziani C Marino M et al. Effect of chronic aspirin therapy on angiographic thrombotic burden in patients admitted for a first ST-elevation myocardial infarction. Am J Cardiol. 2010;105(5):587 591. 10.1016/j.amjcard.2009.10.040) 20185001

[13] TIMI Study Group. The thrombolysis in myocardial infarction (TIMI) trial. Phase I findings. N Engl J Med. 1985;312(14):932 936. 10.1056/NEJM198504043121437) 4038784

[14] Boztosun B Güneş Y Kirma C . Current management of no-reflow. Anadolu Kardiyol Derg. 2006;6(3):255 260. 16943112

[15] Singh M Berger PB Ting HH et al. Influence of coronary thrombus on outcome of percutaneous coronary angioplasty in the current era (the Mayo Clinic experience). Am J Cardiol. 2001;88(10):1091 1096. 10.1016/s0002-9149(01)02040-9) 11703950

[16] Yip HK Chen MC Chang HW et al. Angiographic morphologic features of infarct-related arteries and timely reperfusion in acute myocardial infarction: predictors of slow-flow and no-reflow phenomenon. Chest. 2002 ;122(4):1322 1332. 10.1378/chest.122.4.1322) 12377860

[17] Sardella G Mancone M Bucciarelli-Ducci C et al. Thrombus aspiration during primary percutaneous coronary intervention improves myocardial reperfusion and reduces infarct size: the EXPIRA (thrombectomy with export catheter in infarct-related artery during primary percutaneous coronary intervention) prospective, randomized trial. J Am Coll Cardiol. 2009 ;53(4):309 315. 10.1016/j.jacc.2008.10.017) 19161878

[18] Elgendy IY Huo T Bhatt DL Bavry AA . Is aspiration thrombectomy beneficial in patients undergoing primary percutaneous coronary intervention? Meta-analysis of randomized trials. Circ Cardiovasc Interv. 2015;8(7):e002258. 10.1161/CIRCINTERVENTIONS.114.002258) 26175531

